# Establishment and Application of a Novel Difficulty Scoring System for da Vinci Robotic Pancreatoduodenectomy

**DOI:** 10.3389/fsurg.2022.916014

**Published:** 2022-06-01

**Authors:** Hongfa Sun, Chuandong Sun, Bingyuan Zhang, Kai Ma, Zehua Wu, Brendan C. Visser, Bing Han

**Affiliations:** ^1^ Department of Hepatobiliary and Pancreatic Surgery, The Affiliated Hospital of Qingdao University, Qingdao, China; ^2^ Hepatobiliary & Pancreatic Surgery, Stanford University School of Medicine, Stanford, CA, United States

**Keywords:** robotic surgery, da Vinci, pancreatoduodenectomy, difficulty, difficulty evaluation

## Abstract

**Background:**

Robotic pancreatoduodenectomy (RPD) technology is developing rapidly, but there is still a lack of a specific and objective difficulty evaluation system in the field of application and training of RPD surgery.

**Methods:**

The clinical data of patients who underwent RPD in our hospital from November 2014 to October 2020 were analyzed retrospectively. Univariate and multivariate logistic regression analyses were used to determine the predictors of operation difficulty and convert into a scoring system.

**Results:**

A total of 72 patients were enrolled in the group. According to the operation time (25%), intraoperative blood loss (25%), conversion to laparotomy, and major complications, the difficulty of operation was divided into low difficulty (0–2 points) and high difficulty (3–4 points). The multivariate logistic regression model included the thickness of mesenteric tissue (P1) (*P* = 0.035), the thickness of the abdominal wall (B1) (*P* = 0.017), and the preoperative albumin (*P* = 0.032), and the nomogram was established. AUC = 0.773 (0.645–0.901).

**Conclusions:**

The RPD difficulty evaluation system based on the specific anatomical relationship between da Vinci’s laparoscopic robotic arm and tissues/organs in the operation area can be used as a predictive tool to evaluate the surgical difficulty of patients before operation and guide clinical practice.

## Introduction

Intuitive surgical launched the da Vinci Surgical System in 1999, which was approved by the US FDA for its application in general surgery in 2000. After that, it rapidly developed and expanded into multiple surgical fields. During this period, there have been continuous doubts about robotic surgery’s safety and feasibility, especially for pancreatoduodenectomy, which is a challenging and complicated operation that has many organs removed and reconstructed, and serious postoperative complications may occur. Since the first case of da Vinci robotic pancreatoduodenectomy (RPD) was reported in 2003 (1), its surgical techniques and procedures have been continuously improved and made great progress; in addition to the advantages in digestive tract reconstruction, it is also very convenient for vascular reconstruction.

In order to spread robot technology more safely, a specific difficulty evaluation system is also needed in the field of application and training of RPD surgery. Every surgical operation is not exactly the same, and the clinical evaluation of the difficulty of the operation is also very subjective. In 2014, Ban et al. (2, 3) proposed the DSS-B laparoscopic hepatectomy difficulty scoring system (Ban Difficulty Scoring System), which scores the difficulty of the operation based on five aspects (tumor location, resection scope, tumor size, tumor-vascular relationship, preoperative liver function Child–Pugh Classification) and is divided into three difficulty levels: low, medium, and high. In 2017, Kawaguchi et al. (4, 5) proposed the DSS-ER laparoscopic hepatectomy difficulty score system based on the scope of surgical resection (Difficulty Scoring System Based on Extent of resection), which is a new difficulty evaluation system independent of DSS-B. The main advantage of these evaluation systems is that they can be carried out before operation. Although it is difficult to integrate all the technical difficulties, they are more accurate than subjective evaluation. We believe that such an objective basis can reduce the rate of conversion to laparotomy, shorten unnecessary anesthesia and operation time, and reduce medical injury and blood loss caused by a lack of technical ability. Also, RPD surgery is relatively expensive, so it is imperative to reduce unnecessary conversion to laparotomy, which requires a difficult evaluation system more than ordinary laparoscopic surgery.

The purpose of this study is to design an RPD difficulty evaluation system and name it Han’s Difficulty Scoring System for da Vinci RPD, DSS-Han for RPD. The system can be applied to evaluate the difficulty and probability of success of RPD surgery, reduce the rate of transition to laparotomy, and improve the safety of surgery.

## Methods

### Study Population

All of the patients were consecutive. Include consecutive patients who underwent RPD from November 2014 to October 2020 in our hospital. A total of 72 cases, 42 males and 30 females, with a median age of 61.5 years, were recruited. A complete evaluation included enhanced computed tomography and enhanced magnetic resonance imaging. If the diagnosis was not clear, endoscopic ultrasonography with or without biopsy was performed. We followed the criteria defining the resectability status of NCCN Guidelines for Pancreatic Adenocarcinoma. Inclusion criteria are as follows: (1) ≥ 18 years old; (2) no history of upper abdominal surgery; (3) detailed preoperative imaging data such as enhanced CT or enhanced MRI; and (4) the patient could tolerate general anesthesia (6). Exclusion criteria are as follows: (1) invasion of the common hepatic artery, celiac trunk, or superior mesenteric artery or invasion of the superior mesenteric vein or portal vein; (2) tumor larger than 10 cm; and (3) metastatic disease. Relevant data were collected retrospectively, including baseline patient characteristics, such as demographic data, preoperative risk factors and postoperative complications, types of preoperative management, and surgical characteristics (including intraoperative events, pathological data, and postoperative results). The individuals who collected the data (Luo, Ma, etc.) conducted a detailed review of the medical records to collect baseline demographic and perioperative data. All personnel involved in data collection were required to report on the data collection and audit process to ensure accuracy. The queries based on the omissions, outliers, and differences found in this process were summarized and corrected. All of the operations were performed after obtaining the informed consent of each patient.

### Studied Criteria

#### Surgical Treatment

All of the surgeries were performed by the same group of surgeons (Zhang, Sun, Han). Considering the learning time required for surgeons to develop enough professional level in the implementation of RPD, the surgeons who have completed these operations in this study were all doctors who have done open pancreatoduodenectomy for more than 20 years and have received systematic training in robot operation, their experience was rich, and there was no obvious difference (7, 8). Although our sample size was small, we were accumulating continuously. In China, we belonged to the center with a relatively large scale of da Vinci operation. According to the relevant literature, the learning curve of RPD needed about 30–40 cases (9, 10). Of course, some studies showed that the learning curve needed more than 200 cases (11). Through comparison, it was found that the operation time and intraoperative blood loss of cases in our study were significantly less than those in previous studies (10, 12) (Table 1B). The possible explanation was that we have crossed the early learning curve. Our surgeons have carried out a large number of ordinary laparoscopic pancreaticoduodenectomy, robotic liver lesion resection, robotic distal pancreatectomy, and so on before the complex operation of RPD. Therefore, we had high proficiency in the implementation of RPD at the beginning. However, there is still a certain gap in the long-term learning curve (13), which depends on the further development of robotic surgery and large sample and multicenter research. However, this at least showed that our research has a certain guiding significance at this stage of the development of robotic surgery, which can provide suggestions for the center in the initial and medium stages of robotic surgery.

**Figure 1:**
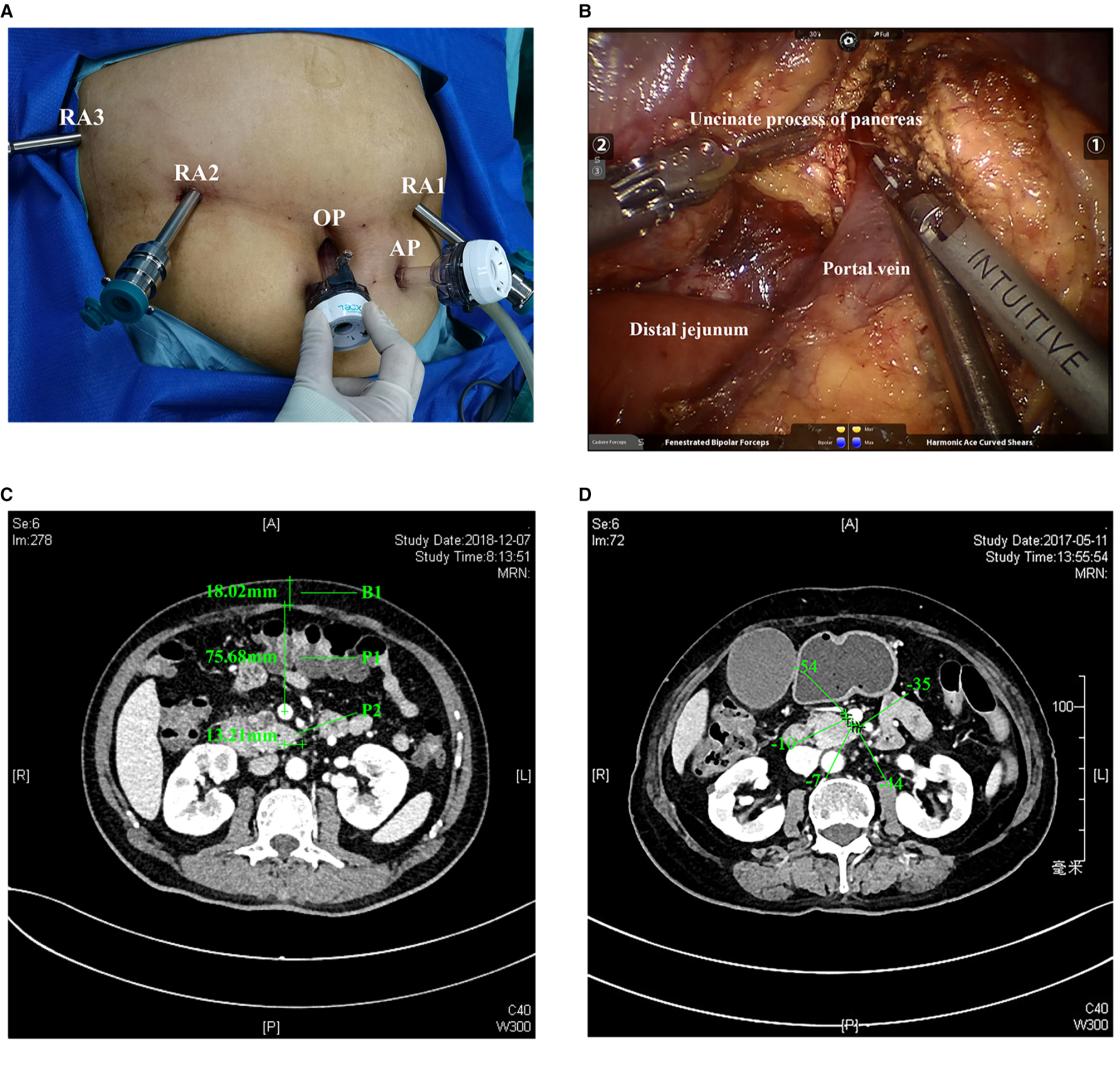
(**A**). Placement of the five ports. RA1, RA2, RA3, the OP, and AP. (**B**). Separation of the uncinate process of the pancreas and portal vein. OP, optic port; AP, assistant port. (**C**). Actual measured P1, P2, and B1 indexes. (**D**). Peripancreatic fat density. The density value is the average of the density where the five plus signs are located in the figure. The CT image is in the venous phase. CT, computed tomography; P1, mesenteric tissue thickness; P2, length of the uncinate process; B1, thickness of the abdominal wall.

RPD used the standard procedures, which were explained in detail in the articles of Jin et al. (14), and was briefly described as follows: used a bottom-up, fixed five-port layout (Figure 1A), including a resection part: RA1, harmonic ace; RA2, fenestrated bipolar forceps; RA3, Cadiere forceps; and a reconstruction part: RA1, large needle driver. The position was head high and foot low, and the angle was 15°–30°. The right side of the patient was raised during the operation of the head of the pancreas, the left side was raised during the operation of the body and tail of the pancreas, and the body lay flat during the middle operation. After exploration, the gastrocolic ligament was first severed. Followed by a Kocher incision, we cut off the jejunum and dissected the portal vein and the neck of the pancreas. Then, the stomach and pancreas were dissected. We amputated the uncinate process of the pancreas (Figure 1B) after dissecting the hepatoduodenal ligament. So far, the specimen was completely severed, followed by pancreaticojejunostomy.

The reserved proximal jejunum was lifted to reconstruct the anastomosis. The team usually performed double pancreaticojejunostomy. Generally, the free broken end was 2 cm. When anastomosing, we carefully look for the main pancreatic duct and place a silicone tube of the suitable size to support the pancreatic duct. 3-0 Prolene was used to suture the seromuscular layer of pancreas–jejunum continuously in the outer ring. The posterior wall was sutured first, and the needle was inserted from the ventral side of the upper edge of the pancreas. The tail line was grasped by Cadiere forceps of RA3, and finally, the needle was inserted from the jejunum of the lower edge of pancreas. When the diameter of the pancreatic duct was ≥3 mm, catheter-to-mucosal reconstruction was the preferred option. A small opening was made in the jejunum mucosa, and the pancreaticojejunostomy was performed with Prolene/PDS6-0 or 5-0 intermittent suture. When the diameter of the pancreatic duct was ≥5 mm, the front and rear walls could be continuously sutured, respectively. After that, the anterior wall of the pancreas–jejunum seromuscular layer was sutured continuously and tied with the caudal line. Next, we performed choledochojejunostomy and gastrojejunostomy, and the last step was to take out the specimen and place the drainage tube.

#### Definition of Variables

Data collection included events during the hospital stay and within 30 days after discharge (15–20). The time of operation was defined as the time from the incision of the skin to the final closure of the skin. The anesthesiologist carefully assessed the blood loss during the operation and recorded it at the end of the procedure. According to the Clavin–Dindo classification, other postoperative complications were determined (21). In addition, delayed gastric emptying (DGE) was taken as one of the criteria to determine the difficulty of surgery in advance when establishing the model because we thought that such an outcome had a significant impact on the hospitalization process of patients, prolonged the length of stay (LOS), and increased the pain of patients. So, we defined complications (Clavin–Dindo ≥ 3) and DGE as major complications as an index to define the difficulty of operation. The diagnosis and classification of postoperative pancreatic fistula were based on the latest International (2016) Pancreatic Fistula Research Group standard (22). Pancreatic leakage of grade B or above were considered meaningful; bleeding and DGE were defined according to the International Pancreatic Surgery Research Group (23, 24); and bile leakage was defined according to the international liver surgery research group’s standards (25). Mesenteric tissue thickness (P1) represented the thickness of mesenteric tissue and was defined as the data obtained by measuring the distance from the midpoint of the portal vein to the anterior peritoneum at the CT level at the tip of the uncinate process. P2 reflected the length of the uncinate process, which was the distance from the tip of the uncinate process of the pancreas to the midpoint of the portal vein at the CT level of the longest uncinate process and was given + or – values according to the position relationship between the uncinate process and the midpoint. B1 represented the thickness of the abdominal wall at the white line of the CT level at the tip of the uncinate process; the dilatation of the pancreatic duct was determined according to the preoperative imaging report (≥3 mm) (26, 27); and the peripancreatic fat density was defined as five points randomly selected from the area between the uncinate process of pancreas and the portal vein at the CT level of the tip of the uncinate process. Then, the average was taken, and the unit was the Hounsfield unit (HU) (28, 29) (Figure 1C,D). Peripancreatic inflammation was defined as follows: due to the stimulation or compression of a lesion, patients often have the experience of pancreatitis. The stimulation of pancreatic juice exudation on the surrounding tissues will cause inflammation and adhesion, which will affect the difficulty of operation. The phenomena of liquid residue and increased tissue density can be observed in imaging (30, 31). All the image data defining these indicators have been saved for reference, if necessary.

#### Definition of Difficulty

The perioperative data reflecting the difficulty of the operation were selected: operation time, intraoperative blood loss, conversion to laparotomy, LOS, and major complications.

These four indexes, namely, three intraoperative indicators of operation time (25%), intraoperative blood loss (25%), and conversion to laparotomy (4) and major complications (19, 32), were evaluated to determine the difficulty of operation. There were certain objectivity because the difficulty of the operation could be reflected as a whole through the combination of these intraoperative and postoperative factors. During the operation time, if blood loss was at or above the 25th percentile of the total cases and conversion to laparotomy or major complications occurred, each RPD operation was assigned 1 point. Therefore, each RPD operation’s score was 0–4 points; 0–2 points were defined as low difficulty, and 3–4 points were defined as high difficulty.

RPD difficulty was divided into two levels: low difficulty and high difficulty. Then, we suggest that RPD should be performed by experienced doctors, while the operation defined as high difficulty should be performed by more experienced doctors (RPD ≥ 10) (2, 4).

### Statistical Analysis

**Figure 2:**
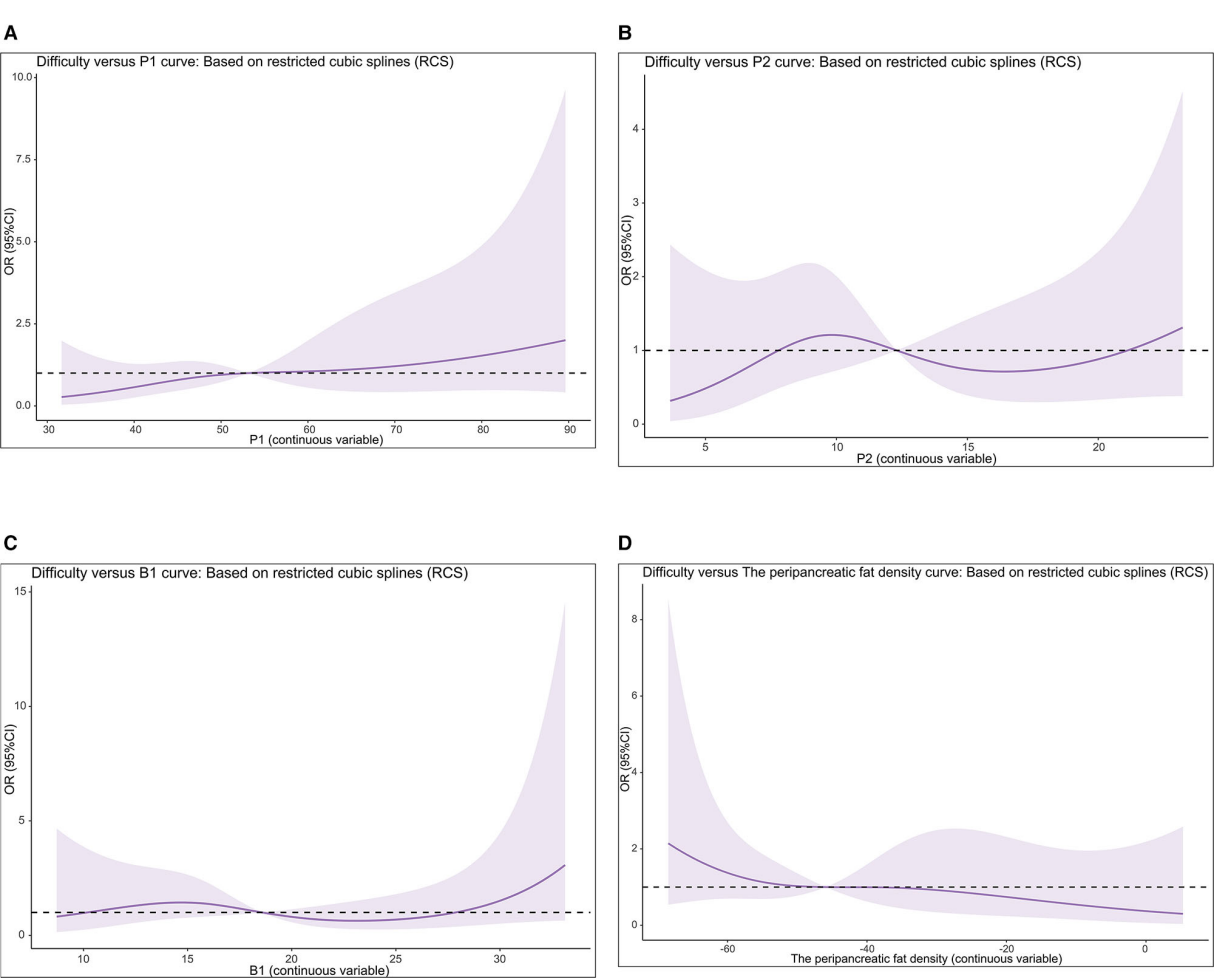
Restricted cubic spline curve.

**Figure 3:**
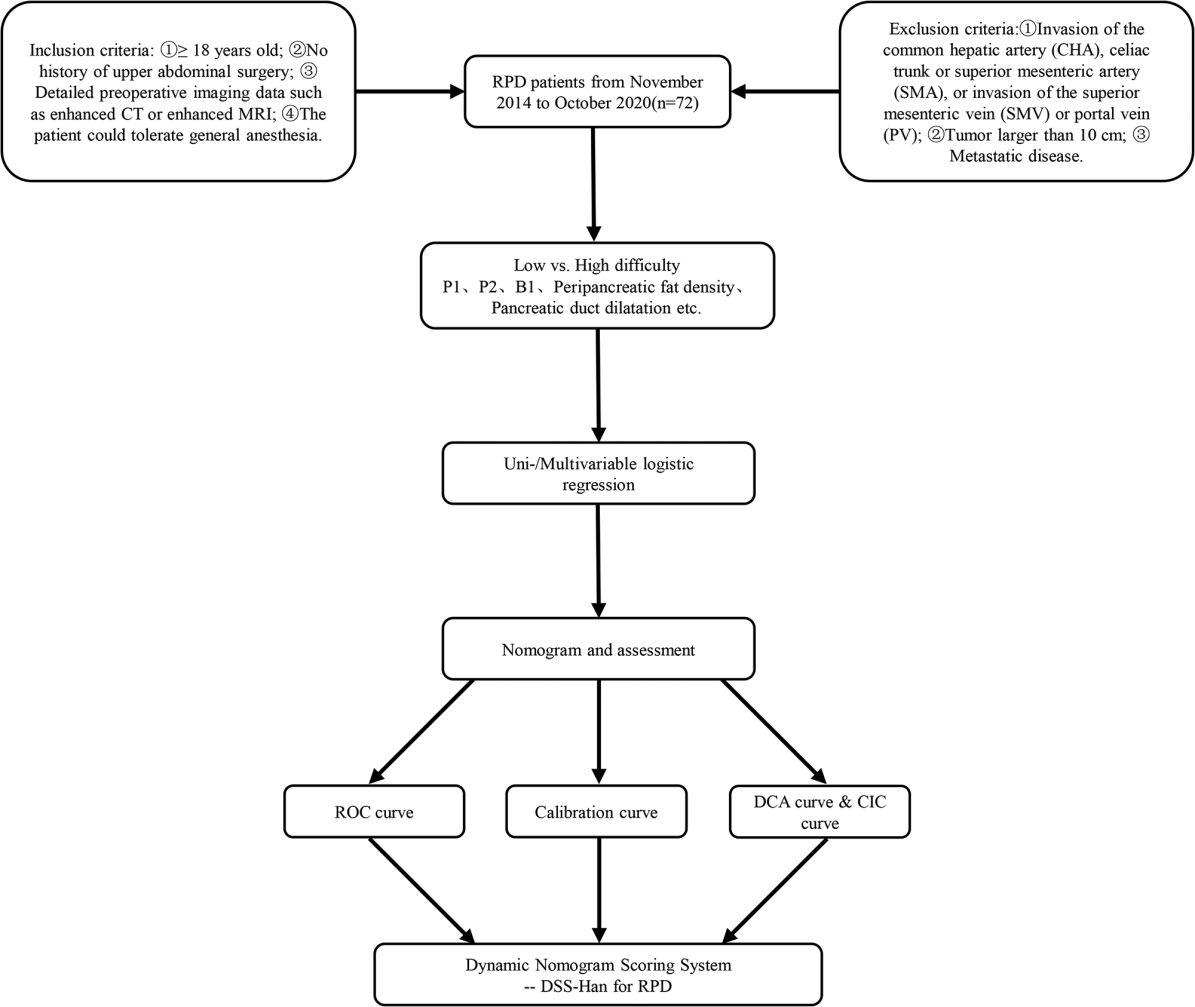
Flow chart of the analysis.

All data used SPSS (version 25.0; IBM Corp, New York) and R software (version 4.0.3; R Development Core Team) for data analysis. Appropriately, in the Shapiro–Wilk normality test, continuous variables were described as mean ± SD or median (interquartile range [IQR]) and tested by the *t*-test or Mann–Whitney *U* test. Categorical variables were represented by numbers (%), passed the *χ*^2^ test, and were tested by the continuity correction of the *χ*^2^ test or Fisher’s exact test. Binary logistic regression was used to build predictive models. The selection of covariates was based on prior validation data (clinical and literature) related to dependent variables or similar clinical outcomes and was combined with univariate analysis and *P*-value. A univariate analysis was performed to examine the relationship between the predefined surgical difficulty and the above variables. We utilized restricted cubic spline (RCS) models/changepoint analysis to determine a clinically meaningful cut point of P1, P2, B1, and the peripancreatic fat density (Figure 2) (33). Independent variables with significant *P* < 0.1 were included in the multivariate analysis, with the difficulty of surgery as the dependent variable. A backward-selected multivariate binary logistic regression model was used to simulate the relationship between candidate patient characteristics, perioperative variables, and surgical difficulty. The Hosmere–Lemeshow goodness-of-fit *χ*^2^ test was used to assess how well the multivariable model fitted the actual data. Also, R software was used to construct a nomogram of covariates and surgical difficulty. Internal verification was evaluated by the area under the curve (AUC) or C-index and the calibration curve. We determined the sensitivity, specificity, and 95% CI of the C-index of this prediction model. Bootstrapping with 1,000 resamples with replacement was used for the calibration curve in order to determine the robustness and accuracy of model prediction performance. We also utilized decision curve analysis (DCA) and the clinical impact curve to evaluate the potential clinical effects of the model. Finally, we developed a web server and scoring system to easily access our new model. In all analyses, two-tailed values of *P* < 0.05 were considered statistically significant. The workflow is shown in Figure 3.

## Results

### Baseline Characteristics

A total of 72 eligible patients were enrolled. Table 1A summarizes the patient characteristics and perioperative details of the study cohort. Among them, there were 42 (58%) males and 30 (42%) females, with a median age of 61.5 (50–67) years. The median operation time was 432.5 (360–503.75) min, with 55 (76%) patients having an operation time above the 25% percentile (360 min); the median blood loss was 200.00 (100.00–200.00) mL, and 62 (86%) patients had an intraoperative blood loss above the 25% percentile (100 mL); 4 (6%) patients converted to laparotomy, and there were 20 (27.8%) cases that occurred postoperative complications (Clavien–Dindo grade ≥3) and 33 (46%) cases that occurred major complications. Finally, 49 (68%) patients were defined as low difficulty and 23 (32%) cases as high difficulty.

**Table 1A:** Baseline perioperative characteristics.

Baseline variables	Total	Low difficulty	High difficulty	*P*
Number	72 (100)	49 (68)	23 (32)	–
Gender, male	42 (58)	26 (53)	16 (70)	0.185^c^
Age, years	61.5 (50–67)	60.00 (46.50–66.00)	65.00 (54.00–68.00)	0.150^b^
BMI, kg/m^2^*	23.53 ± 2.88	23.28 ± 2.91	24.07 ± 2.79	0.282^a^
Jaundice, yes	43 (60)	30 (61)	13 (57)	0.704^c^
WBC, 109/L**	5.82 (4.61–6.80)	5.99 (4.80–7.06)	5.43 (3.89–6.60)	0.136^b^
Preoperative albumin, g/L*	40.04 ± 3.90	40.63 ± 3.65	38.79 ± 4.20	0.062^a^
Preoperative total bilirubin, μmol/L**	65.79 (15.44–205.71)	72.66 (14.16–219.45)	50.05 (15.65–183.20)	0.677^b^
Blood glucose, mmol/L**	5.55 (4.81–6.28)	5.52 (4.83–6.24)	5.73 (4.74–7.32)	0.726^b^
P1, mm*	54.47 ± 17.92	51.60 ± 17.24	60.58 ± 18.17	0.047^a^
P1 (> 53.16 mm)	36 (50)	20 (41)	16 (70)	0.023 ^c^
P2, mm*	12.53 ± 6.78	11.86 ± 6.47	13.97 ± 7.34	0.221^a^
P2 (<7.8 mm)	23 (32)	18 (37)	5 (22)	0.203 ^c^
B1, mm*	19.85 ± 7.45	19.32 ± 7.05	20.97 ± 8.29	0.383^a^
B1 (>27.9 mm)	12 (17)	5 (10)	7 (30)	0.032 ^c^
Peripancreatic fat density, HU**	−45.90 (−56.55–27.60)	−44.40 (−55.60–15.60)	−54.00 (−58.20–31.60)	0.079^b^
Peripancreatic inflammation, yes	10 (14)	7 (14)	3 (13)	0.887^c^
Hospitalization expenses (RMB)**	127,970.00 (110,961.75–150,879.39)	115,965.97 (106,193.00–133,211.41)	153,216.55 (136,387.24–204,939.00)	0.000^b^
Pancreatic duct dilatation, yes	30 (42)	24 (49)	6 (26)	0.066^c^
Adenocarcinoma, yes	48 (67)	31 (63)	17 (74)	0.372^c^
Vascular invasion, yes	17 (24)	12 (24)	5 (22)	0.798^c^
Operative time, min*	434.71 ± 99.55	411.67 ± 94.03	483.78 ± 94.83	0.003^a^
Operative time (≥360 min)	55 (76)	33 (67)	22 (96)	0.008^c^
Intraoperative bleeding, mL**	200.00 (100.00–200.00)	150.00 (100.00–200.00)	200.00 (150.00–300.00)	0.017^b^
Intraoperative bleeding (≥100 mL)	62 (86)	39 (80)	23 (100)	0.020^c^
Conversion to laparotomy, yes	4 (6)	1 (2)	3 (13)	0.057^c^
Length of stay(total), day**	24.00 (18.00–36.00)	21.00 (17.00–26.50)	37.00 (25.00–43.00)	0.000^b^
Length of stay, day**	15.00 (11.00–29.00)	12.00 (10.00–17.00)	30.00 (19.00–38.00)	0.000^b^
Complications (≥3)	20 (28)	8 (16)	12 (52)	0.002^c^
Major complications, yes	33 (46)	10 (20)	23 (100)	0.000^c^
Delayed gastric emptying, yes	16 (22)	5 (10)	11 (48)	0.000^c^
Perioperative death, yes	3 (4)	0 (0)	3 (13)	0.010^c^

*Abbreviations: P1, mesenteric tissue thickness; P2, length of the uncinate process; B1, thickness of the abdominal wall; BMI, body mass index; WBC, white blood cell.*

^a^ *Student’s t-test.*

^b^ *Mann–Whitney U test.*

^c^ *Pearson’s χ^2^ test.*

Values in parentheses are N (%) unless indicated otherwise; values are *mean(s.d.) and **median (i.q.r.).

**Table 2:** Comparison of operation outcome indicators.

Author-year	*N* = No. of patients	Operation time, min	Intraoperative blood loss, mL
Current study**	*N* = 72	432.50 (360.00–503.75)	200.00 (100.00–200.00)
Watkins-2017**	*N* = 92	504.00 (438.00–570.00)	242.00 (43.00–441.00)
Napoli-2016*	*N* = 70	564.00 ± 101.70	–

*Values are* *mean (s.d.) and *median (i.q.r.).

The indexes related to operation time (average ± standard deviation, median (IQR), 25% percentile) (*P* < 0.01), blood loss (median (IQR), 25% Percentile) (*P* < 0.05), and the LOS (total days, postoperative days) (*P* < 0.001) of the low-difficulty group were significantly lower than those of the high-difficulty group. Complication-related outcomes of the low-difficulty group were significantly lower than those of the high-difficulty group [complications (Clavien–Dindo grade ≥3) and DGE (*P* < 0.01), pancreatic leakage (*P* < 0.001), abdominal infection (*P* < 0.01), intestinal leakage (<0.05)]. Three cases died during the perioperative period, and all of them were in the high-difficulty group (*P* < 0.05). P1(mean) was 54.47 ± 17.92 mm, and the low-difficulty group was smaller than the high-difficulty group (51.60 vs. 60.58 (average), *P* < 0.05); B1(mean) was 19.85 ± 7.45 mm, and low vs. high = 19.32 vs. 20.97 (mean) (*P* = 0.383); B1 (> 27.9 mm) defined by the RCS method: low vs. high = 10% vs. 30% (*P* = 0.039); the hospitalization cost (median) is 127,970.00 (110,961.75–150,879.39) RMB, and the low-difficulty group was smaller than the high-difficulty group (115,965.97 vs. 153,216.55 (median), *P* < 0.01).

P2(mean) was 12.53 ± 6.78 mm, low-difficulty group vs. high-difficulty group = 11.86 vs. 13.97 (mean). The peripancreatic fat density was −45.90 (−56.55–27.60); 36 (50%) cases were above the median, low vs. high = −44.40 vs. −54.00. Ten (14%) cases had peripancreatic inflammation. Although the above data were not statistically significant (*P* > 0.05), clinicians confirmed it in clinical practice generally. Additional patient characteristics can be obtained from Supplementary Table.

### Construction and Assessment of Nomogram

**Figure 4:**
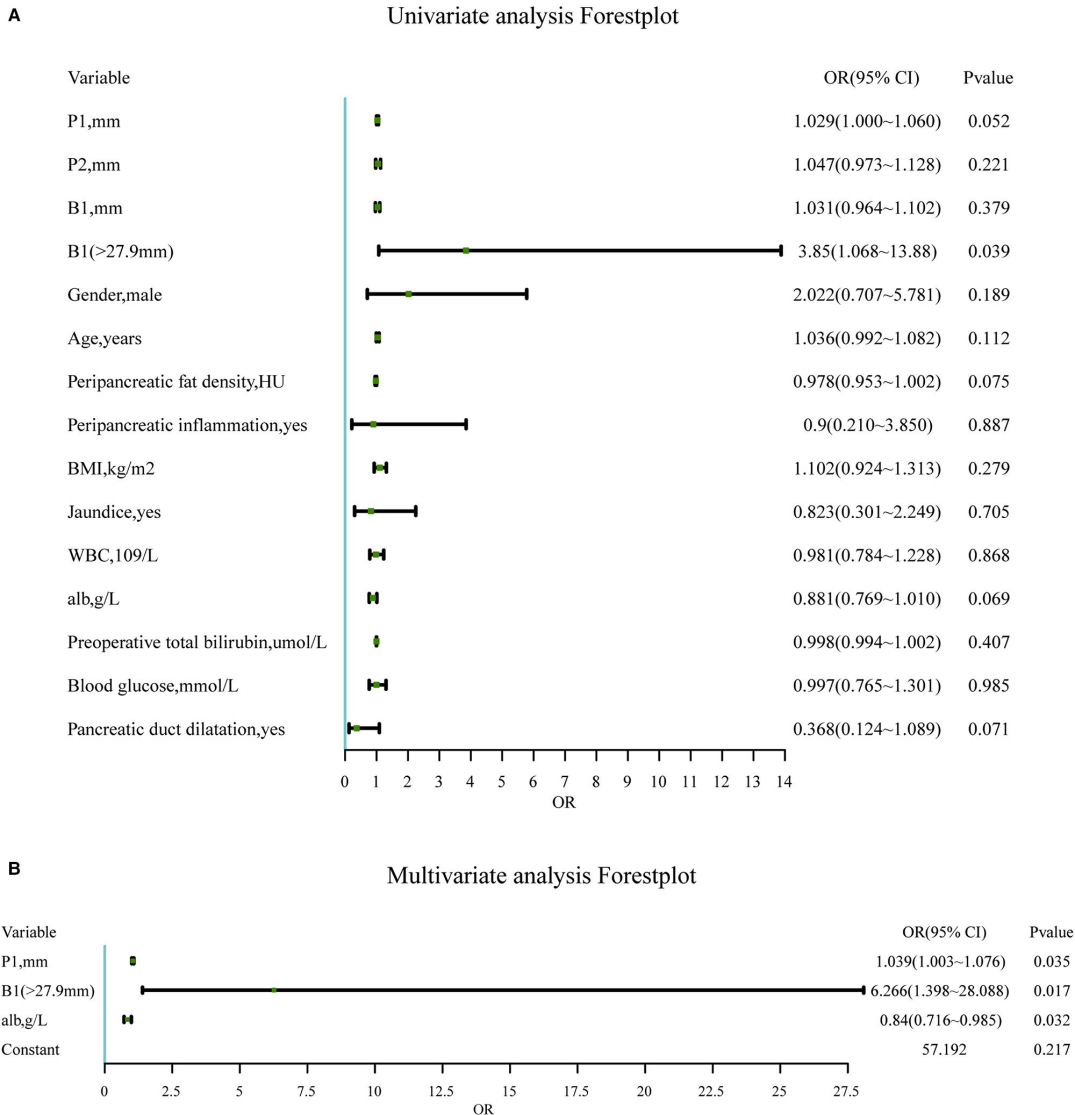
(**A**). Univariate analysis of candidate predictors associated with the difficulty of RPD surgery. *Statistically significant (univariate binary logistic regression). (**B**). Significantly independent multivariable predictors of the difficulty of RPD surgery. The multivariable logistic regression model is adjusted for the site. Abbreviations: P1, mesenteric tissue thickness; P2, length of the uncinate process; B1, thickness of the abdominal wall; BMI, body mass index; WBC, white blood cell.

Several variables related to surgical difficulty were determined by univariate logistic regression (Figure 4A). There was statistical significance in P1, B1 (> 27.9 mm), the peripancreatic fat density, the preoperative albumin, and whether the pancreatic duct was dilated, which might affect the difficulty of surgery.

In the final multivariate logistic regression model (Figure 4B), independent predictors of surgical difficulty included P1 (*P* < 0.05), B1 (> 27.9 mm) (*P* < 0.05), and the preoperative albumin (*P* < 0.05). The dilated pancreatic duct was excluded due to failure when included in the multivariate model (*P* = 0.102).

Model estimation and testing utilized the maximum likelihood method. The goodness-of-fit test (Hosmere–Lemeshow) confirmed a good model fit to the data (*χ*^2^ = 7.115 on eight degrees of freedom; *P* = 0.524) and showed no significant differences between the surgical difficulty predicted by this model and the measured surgical difficulty.

**Figure 5:**
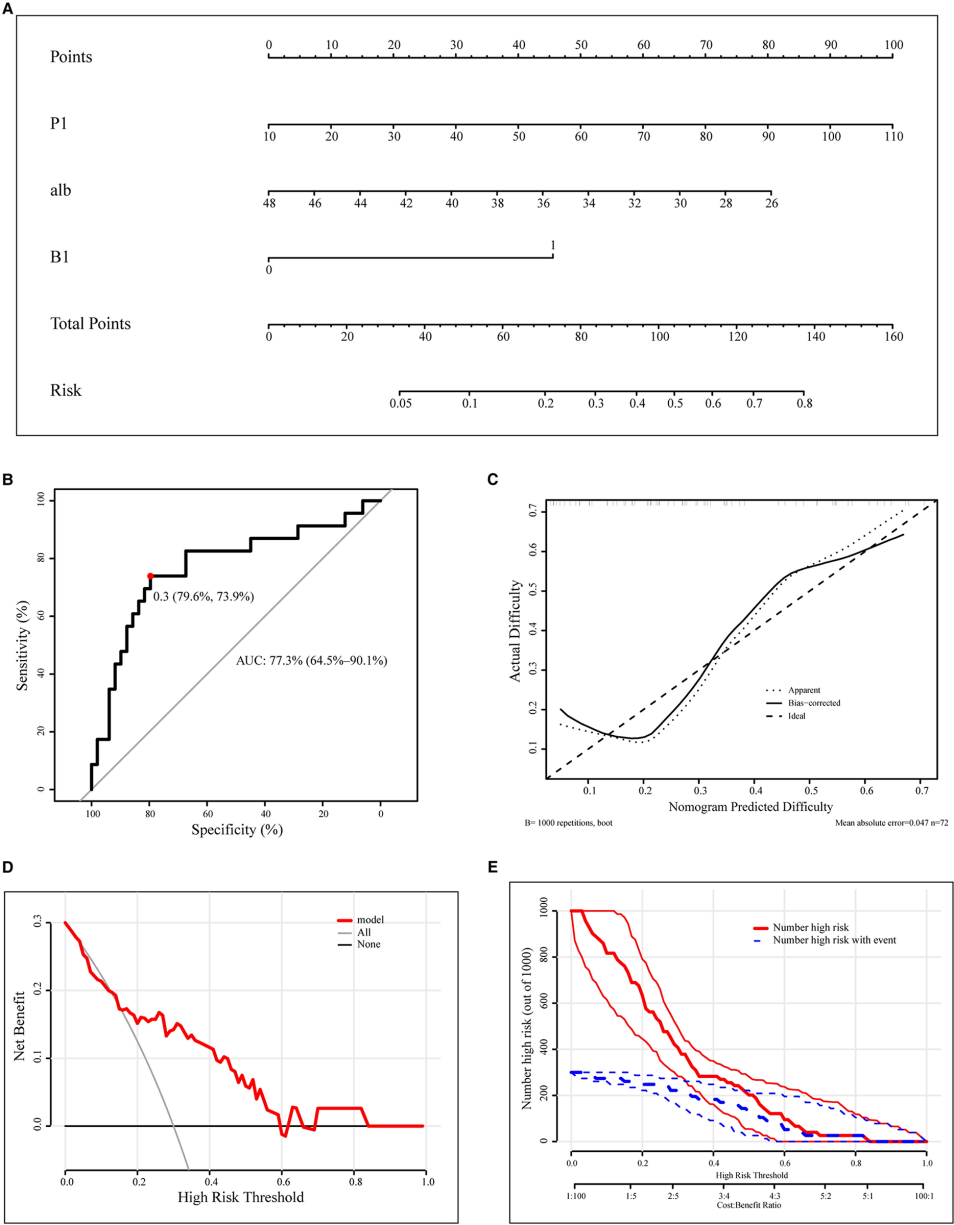
Construction and calibration of nomograms for RPD that integrates pathological features and anatomical factors. (**A**). Development of the nomogram to predict the difficulty of RPD. (**B**). ROC curve of the nomogram model. (**C**). Calibration curve of the nomogram. (**D**). DCA curve. (**E**). CIC curve. RPD, robotic pancreatoduodenectomy; ROC, receiver operating characteristic curve; DCA, decision curve analysis; CIC, clinical impact curve; alb, preoperative albumin.

**Figure 6:**
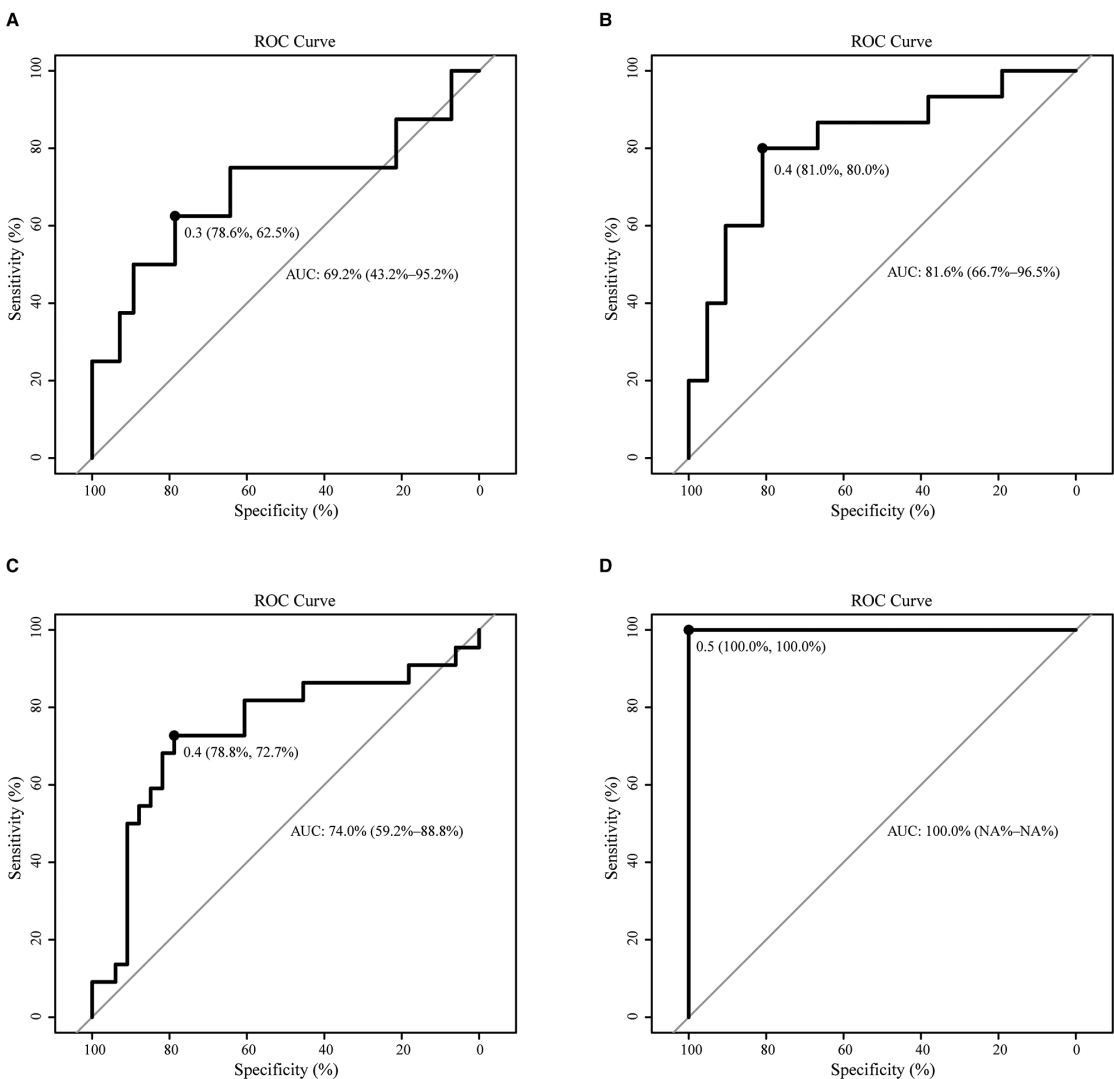
Subgroup validation of the model. (**A**). ROC of the early cohort (*N* = 36); (**B**). ROC of the late cohort (*N* = 36); (**C**). ROC of the long-time cohort (*N* = 55); (**D**). ROC of the short-time cohort (*N* = 17).

In order to predict the difficulty of operation in patients with RPD, a prediction model of surgical difficulty, including independent risk factors, was established (Figure 5A). The ROC curve of the model is shown in Figure 5B. We used the C-index or AUC to judge that the model had a good prediction accuracy (0.773; 95% CI: 0.645–0.901). Moreover, the recommended optimal threshold probability is 0.3 when the specificity of the model is 79.6% and the sensitivity is 73.9%. The calibration curve of the predicting model (Figure 5C) showed a good agreement between the nomogram prediction and the actual value. Besides, the bootstrap validation of the multivariate logistic regression model (using logistic regression based on 1,000 resamples in R) confirmed good internal validity. The Brier probability score was low and close to 0 (0.170). The DCA curve (34) showed that in a large range of threshold probability, the benefit of the model was higher than that of the extreme curve, and the net benefit could be calculated, so we could choose a larger range of threshold probability, which was relatively safer (Figure 5D). The next function was the further development of the DCA algorithm by Kerr et al. (35), which was drawing the clinical impact curve, which could help us to choose the threshold probability to achieve the highest clinical effectiveness (Figure 5E). We divided patients into an early cohort and late cohort (36:36) according to the chronological order of patient inclusion. The ROC curve showed that the model had good performance in early and late cohorts (Figures 6A,B). We divided patients into a long-time cohort and a short-time cohort according to 25% of operation time (55:17). The ROC curve also showed that the model had good performance in long-time and short-time cohorts (Figures 6C,D).

## Discussion

The surgery process has experienced from traditional open surgery to laparoscopic surgery; hand-assisted laparoscopic surgery also provides help to improve the prognosis of patients (36). At present, robotic surgery has made great progress (37). Pancreatoduodenectomy (PD) is the only potentially curative treatment for periampullary cancer and premalignant lesions. Unfortunately, only a few patients are candidates for PD at the time of diagnosis. Patients with advanced stage can choose palliative surgery, such as hepatico- and gastrojejunostomy (38–40). In order to further explore the feasibility of RPD and spread robotic technology more safely (7, 8, 41), we developed an RPD difficulty evaluation system based on the specific anatomical relationship between da Vinci’s laparoscopic robotic arm and tissues/organs in the operation area, which can be used as a predictive tool to evaluate the surgical difficulty of patients before operation and guide clinical practice. Then, we used R software to include four independent predictors in the nomogram, which can be used as a repeatable and convenient model to predict the difficulty of RPD surgery. From November 9, 2020 to May 10, 2021, a small validation sequence of five cases of RPD in our center showed that the accuracy rate of predicting the difficulty of operation was 100%, except one case that was excluded due to previous abdominal surgery. We think this is good evidence of the applicability of this model.

The discussion of our study mainly focused on several indicators, which were based on the specific anatomical relationship between da Vinci’s laparoscopic robotic arm and tissues/organs in the operation area. We first proposed and applied them to judge the difficulty of RPD surgery.

In our study, we first propose DSS-Han for the RPD system, which is based on perioperative clinicopathological factors to predict the difficulty of RPD surgery. No one else has done similar research in RPD before. The operation time, intraoperative blood loss, conversion to laparotomy rate, and complications (Clavien–Dindo grade ≥ 3) in this study are similar to those reported in other single-center or multicenter reports (32, 42, 43), so our DSS-Han for the RPD system may also be comparable in other institutions. We first discover and propose the concept of mesenteric tissue thickness (P1) in clinical practice. If the value of P1 is too large, the da Vinci Arm #1 robotic arm is farther from the uncinate process and portal vein, and it will be limited in the visual field, operation angle, and flexibility, which will directly increase the difficulty of the operation. In DSS-Han for the RPD system, P1 determined and verified by multivariate logistic regression was an independent risk factor for the difficulty of RPD operation. The larger the P1, the more difficult the operation (Supplementary 1A). In addition, the larger the + value of the length of the uncinate process (P2), the more difficult the uncinate process resection. P2 (mean) was 12.53 ± 6.78 mm, including the + value in 70 cases and the – value in 2 cases. The average length of P2 in the high-difficulty group was 2.11 mm longer than that in the low-difficulty group, and there was no statistical difference (*P* = 0.221) (Supplementary 1B). It may be related to the small sample size, but according to the intraoperative experience of surgeons, the larger the uncinate process, the more difficult it is to remove, which is more obvious under the endoscope. Because there is no muscle at the white line, the value obtained mainly depends on the thickness of abdominal fat (B1), so this index is similar to BMI and can objectively reflect the nutritional status of patients. According to surgical experience, the difficulty of operation is also related to the degree of obesity of patients. The average length of B1 was 19.85 ± 7.45 mm. The average length of B1 in the high-difficulty group was 1.65 mm longer than that in the low-difficulty group (*P* = 0.383); B1 (>27.9 mm) (low vs. high = 10% vs. 30%) (*P* = 0.039) was an independent risk factor for the difficulty of RPD operation (Supplementary 1D).

In addition, the peripancreatic fat density (28, 29) reflects the degree of pancreatic inflammation. We believe surgeons have the experience that the more severe the inflammation of the pancreas, the more difficult it is to separate the space between the pancreas and the portal vein, but this kind of pancreatic tissue is relatively tough (15–17) and is easy to anastomose. It is not easy to have postoperative pancreatic leakage. The peripancreatic fat density (median) was −45.90 (−56.55–27.60). The density of the high-difficulty group was 9.6 lower than that of the low-difficulty group (*P* < 0.079 < 0.1) (Supplementary 1C). After statistical analysis, combined with the situation during the operation, the dilatation of the pancreatic duct (26, 27) is also an important factor affecting the difficulty of the operation. Interestingly, although dilated pancreatic ducts are often accompanied by pathological factors, such as inflammation, dilated pancreatic ducts are more easily anastomosed during the operation, which can shorten the operation time and reduce the probability of postoperative complications such as pancreatic leakage. The pancreatic duct dilatation rate of the low-difficulty group was 49%, and that of the high-difficulty group was 26% (*P* = 0.066 < 0.1) (Supplementary 1E). The above indicators are found to be of practical significance in clinical practice, which we think are helpful in evaluating the difficulty of operation.

The level of albumin before the operation is closely related to the process of operation and postoperative recovery. Low albumin can cause tissue edema and fragility. It has been made clear by other studies before (44–46). Our study confirms that patients’ preoperative albumin is a protective factor.

With the progress of surgical technology, the incidence of complications caused by minimally invasive surgery has also decreased significantly (47, 48). In RPD surgery, there were many kinds of postoperative complications, among which the incidence rate of pancreatic leakage was high (15, 16, 18), but the serious consequences (≥B grade) were relatively low. Postoperative gastroparesis could significantly prolong the LOS (without DGE vs. with DGE = 12.00 (10.25–17.75) vs. 37.00 (29.25–54.00), *P* < 0.001). Complications (without complications vs. with complications = 115,172 (105,570.32–127,526) vs. 151,796 (133,455.30–227,445.27), *P* < 0.001) and hospitalization days (Pearson’s *r* = 0.736, *P* < 0.001) were also the main influencing factors of hospitalization cost (Supplementary 2). Reasonable evaluation of the difficulty of operation could improve the utilization of resources and reduce the cost to patients and the healthcare system. As a complex surgical method, RPD needs to be discussed in all aspects to help clinicians make decisions and benefit more patients.

## Limitation

Our research also has some limitations. First, this is a retrospective study that uses a combination of intraoperative and postoperative outcomes to classify the difficulty of RPD, and we validate it by evaluating preoperative factors, some of which are related to each other. Since the time of operation, estimated blood loss, and conversion to laparotomy may be affected by the skills of surgeons, they may not be applicable to every surgeon. Second, as with any observed data set, although multivariate analysis is used to determine independent predictors, causality cannot be established. Third, all cases are from patients who underwent RPD surgery in the Affiliated Hospital of Qingdao University, and the sample size is relatively small; there is a sample deviation, so it is absolutely necessary to conduct an external verification test, and further research should be carried out in a multicenter and expected environment in the future. Fourth, all of the cases included are lesions in the lower segment of the bile duct, the head of the pancreas, or the duodenum. The maximum diameter of the tumor was 8.2 cm. All of the cases are evaluated by experience that the da Vinci Surgical System can complete the surgical resection. However, the risk factors we selected are mainly affected by anatomical relationships, while the biological behaviors of different diseases have little impact on these risk factors.

## Conclusion

In a word, around the specific anatomical relationship between da Vinci’s laparoscopic robotic arm and tissues/organs in the operation area, we propose a new difficulty evaluation system, which mainly focuses on the quantitative and objective evaluation of the normal and pathological anatomical factors related to the uncinate process of the pancreas and the portal vein. It can be measured conveniently before the operation and guide clinical practice. It is hoped that this result will contribute to the safer promotion and application of da Vinci robot technology and ultimately benefit more patients.

## Data Availability

The original contributions presented in the study are included in the article/Supplementary Material; further inquiries can be directed to the corresponding author/s.
