# Supplementary figures and images for: Establishment and Application of a Novel Difficulty Scoring System for da Vinci Robotic Pancreatoduodenectomy
Source: Front Surg. 2022 Jun 1;9:916014. doi: 10.3389/fsurg.2022.916014 (PMC9200290; doi:10.3389/fsurg.2022.916014)

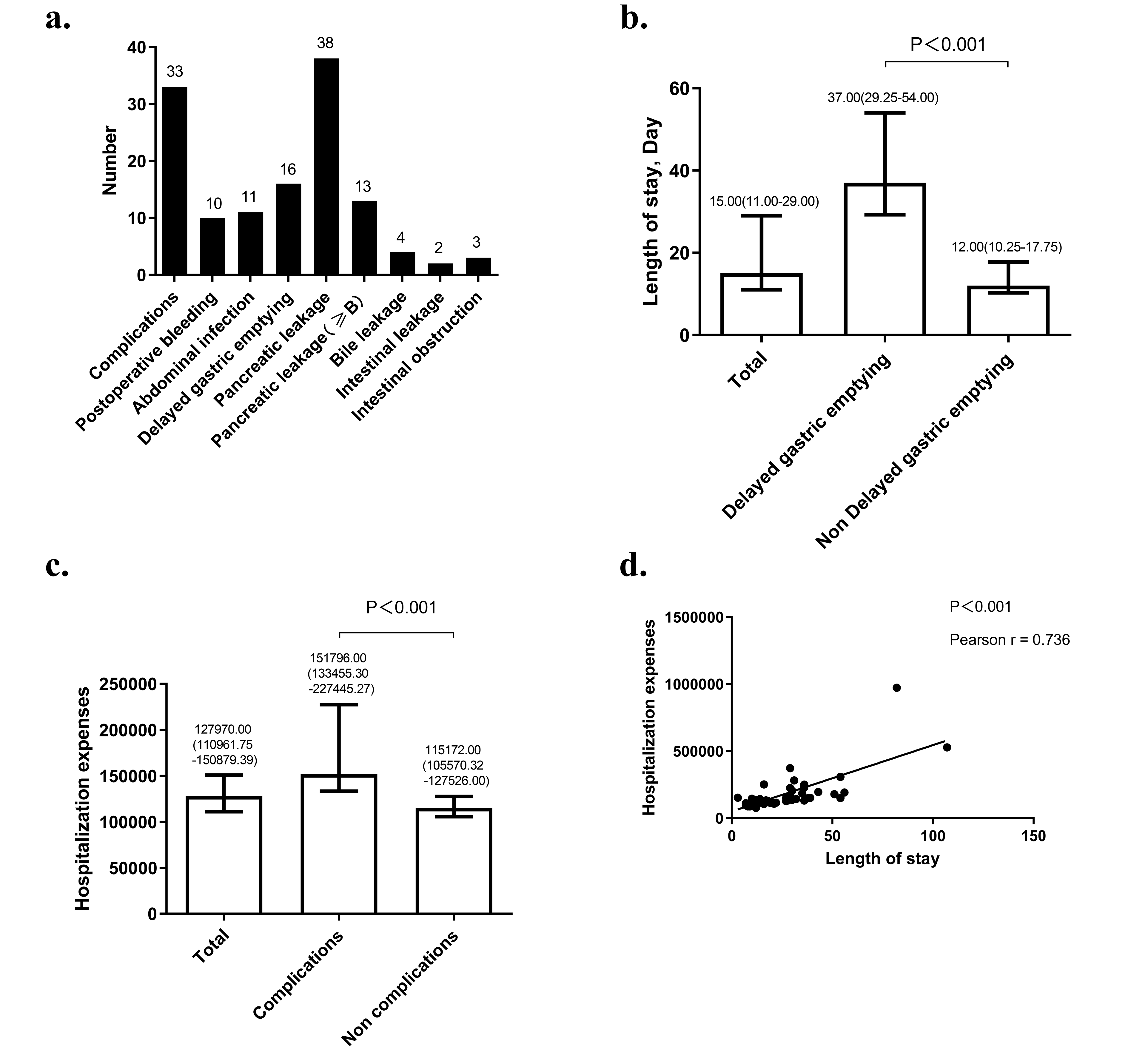

Supplement: Supplementary file 2 [file Image_2_v1.tif]
