# Supplementary material for: Establishment and Application of a Novel Difficulty Scoring System for da Vinci Robotic Pancreatoduodenectomy
Source: Front Surg. 2022 Jun 1;9:916014. doi: 10.3389/fsurg.2022.916014 (PMC9200290; doi:10.3389/fsurg.2022.916014)
Supplement: Supplementary file 3 [file Table_1_v2.docx]

Supplement table.

| Baseline variables | Total | Low difficulty | High difficulty | P |
| --- | --- | --- | --- | --- |
| Symptoms,yes | 62(86) | 42(86) | 20(87) | 0.887^c^ |
| Basic diseases,yes | 39(54) | 26(53) | 13(57) | 0.783^c^ |
| BMI(≤18.5 or ＞28kg/m2) | 14(19) | 9(18) | 5(22) | 0.736^c^ |
| Weight change,yes | 23(32) | 16(33) | 7(30) | 0.851^c^ |
| Neutrophils,109/L^**^ | 3.39(2.49-4.37) | 3.50(2.59-4.71) | 2.72(2.18-4.03) | 0.211^b^ |
| Neutrophils(2~7*109/L) | 11(15) | 7(14) | 4(17) | 0.733^c^ |
| Lymphocyte,109/L^*^ | 1.62±0.59 | 1.65±0.58 | 1.55±0.63 | 0.517^a^ |
| Lymphocyte(0.8~4*109/L) | 3(4) | 2(4) | 1(4) | 0.958^c^ |
| Platelets,109/L^*^ | 250.46±82.56 | 251.10±75.87 | 249.09±97.15 | 0.924^a^ |
| Platelets(100~300*109/L) | 18(25) | 12(24) | 6(26) | 0.884^c^ |
| Preoperative albumin(40~55g/L) | 36(50) | 21(43) | 15(65) | 0.077^c^ |
| Preoperative total bilirubin(＞34.2) | 43(60) | 30(61) | 13(57) | 0.704^c^ |
| Preoperative total bilirubin(≥300) | 4(6) | 3(6) | 1(4) | 0.759^c^ |
| Glutamyl transpeptidase,U/L^**^ | 373.00(26-926.50) | 377.00(26.00-976.50) | 371.00(31.00-899.00) | 0.731^b^ |
| Glutamyl transpeptidase(11~50U/L) | 49(68) | 33(67) | 16(70) | 0.851^c^ |
| Alkaline phosphatase,U/L^**^ | 276.00(84.50-439.50) | 291.00(94.50-504.50) | 215.00(64.00-343.00) | 0.093^b^ |
| Alkaline phosphatase(45~125U/L) | 46(64) | 33(67) | 13(57) | 0.373^c^ |
| Triglyceride,mmol/L^**^ | 1.64(0.86-2.57) | 1.50(0.88-2.46) | 1.75(0.85-2.95) | 0.398^b^ |
| Triglyceride(0.56~1.70mmol/L) | 36(50) | 24(49) | 12(52) | 0.800^c^ |
| Total cholesterol,mmol/L^**^ | 5.50(4.68-6.79) | 5.32(4.43-6.64) | 5.59(5.05-7.12) | 0.200^b^ |
| Total cholesterol(2.9~6.0mmol/L) | 26(36) | 16(33) | 10(43) | 0.373^c^ |
| High-density lipoprotein,mmol/L^**^ | 1.11(0.59-1.47) | 1.19(0.59-1.47) | 1.06(0.69-1.49) | 0.986^b^ |
| High-density lipoprotein(0.94~2.0mmol/L) | 32(44) | 22(45) | 10(43) | 0.910^c^ |
| Low-density lipoprotein,mmol/L^**^ | 3.52(2.71-4.32) | 3.25(2.40-4.42) | 3.64(2.91-4.25) | 0.239^b^ |
| Low-density lipoprotein(2.07~3.12mmol/L) | 44(61) | 28(57) | 16(70) | 0.313^c^ |
| Blood glucose(3.9~6.1mmol/L) | 27(38) | 16(33) | 11(48) | 0.215^c^ |
| Peripancreatic fat density(-41HU) | 29(40) | 23(47) | 6(26) | 0.093^c^ |
| Peripancreatic fat density(-45.9HU) | 36(50) | 27(55) | 9(39) | 0.206^c^ |
| Operative time,min^**^ | 432.5（360-503.75） | 406.00（341.00-485.00） | 500.00（400.00-525.00） | 0.006^b^ |
| Postoperative bleeding,yes | 10(14) | 5(10) | 5(22) | 0.187^c^ |
| Abdominal infection,yes | 11(15) | 3(6) | 8(35) | 0.002^c^ |
| Pancreatic leakage,yes | 13(18) | 3(6) | 10(43) | 0.000^c^ |
| Bile leakage,yes | 4(6) | 1(2) | 3(13) | 0.057^c^ |
| Postoperative intestinal leakage,yes | 2(3) | 0(0) | 2(9) | 0.036^c^ |
| Intestinal obstruction,yes | 3(4) | 3(6) | 0(0) | 0.225^c^ |
| Pancreatic leakage(BL),yes | 38(53) | 24(49) | 14(61) | 0.346^c^ |

Values in parentheses are N (%) unless indicated otherwise; values are ^*^mean(s.d.) and ^**^median (i.q.r.).

^a^ The Student t test.

^b^ Mann‐Whitney U test.

^c^ Pearson's χ2 test.

Abbreviations:P1, mesenteric tissue thickness;P2, the length of the uncinate process; B1, the thickness of the abdominal wall; BMI, body mass index; WBC, white blood cell.
