# Supplementary material for: Establishment and Application of a Novel Difficulty Scoring System for da Vinci Robotic Pancreatoduodenectomy
Source: Front Surg. 2022 Jun 1;9:916014. doi: 10.3389/fsurg.2022.916014 (PMC9200290; doi:10.3389/fsurg.2022.916014)
Supplement: Supplementary file 6 [file Table_4_v1.docx]

**April 8th, 2022**

Dear editor,

We are submitting the manuscript entitled “**Establishment and application of a novel difficulty scoring system for Davinci Robotic Pancreatoduodenectomy.**” by Sun et al, for consideration for publication as a research paper in “**Frontiers in Surgery**”. We believe this study will be of significant interest to many readers of “**Frontiers in Surgery**”.

According to the time of operation, intraoperative blood loss, conversion to laparotomy and major complications, in this study the difficulty of operation was divided into two categories: Low and high difficulty. We used R software to include independent predictors in the nomogram, which can be used as a repeatable and convenient model to predict the difficulty of RPD surgery. Nomograms are a reliable statistical model that build graphical prediction tools by combining all the risk factors of surgical difficulty, and provides a personalized, evidence-based and highly accurate risk assessment. Regarding the specific anatomical relationship between Davinci's laparoscopic robotic arm and tissues/organs in the operation area, we propose a novel difficulty evaluation system, which primarily focuses on the quantitative and objective evaluation of the normal and pathological anatomical factors related to the uncinate process of the pancreas and the portal vein. It is hoped that this result will contribute to the safer promotion and application of the Davinci robot technology, and ultimately benefit more patients.

This article is original, is not under consideration and has not been previously published elsewhere, and will not be submitted elsewhere while acceptance by “**Frontiers in Surgery**” is under consideration. All authors of this paper have directly participated in the planning, execution, or analysis of the study. All authors of this paper have read and approved the final version submitted. The authors also declare that there are no competing financial interests. The key point of our study mainly focused on several indicators, which were based on the specific anatomical relationship between Davinci's laparoscopic robotic arm and tissues/organs in the operation area. We first proposed and applied them to judge the difficulty of RPD surgery. That's why we contributed.

Thank you for your kind consideration of this manuscript, and we look forward to your response.

Sincerely Yours,

Hongfa Sun
